# Mechanism governing the formation of atomically precise dithiolate-protected gold nanoclusters

**DOI:** 10.1039/d6sc00460a

**Published:** 2026-03-13

**Authors:** Sara Yoshikawa, Tokuhisa Kawawaki, Sakiat Hossain, Yuichi Negishi

**Affiliations:** a Institute of Multidisciplinary Research for Advanced Materials, Tohoku University Katahira 2-1-1, Aoba-ku Sendai 980-8577 Japan tokuhisa.kawawaki.d8@tohoku.ac.jp yuichi.negishi.a8@tohoku.ac.jp; b Carbon Value Research Center, Research Institute for Science and Technology, Tokyo University of Science Kagurazaka Shinjuku-ku Tokyo 162-8601 Japan

## Abstract

Atomically precise metal nanoclusters (NCs) stabilized by organic ligands are promising functional materials in various fields owing to their unique geometric and electronic structures. However, many such NCs exhibit insufficient stability, *e.g.*, processes such as alloying can induce structural destabilization. Gold (Au) NCs can be protected by introducing multi-site thiolates (SR), which form exceptionally strong Au–S bonds, thus enhancing the stability of the NCs and expanding their practical applicability. However, multi-site SR protection using bidentate ligands often leads to undesirable polymerization due to inter-NC cross-linking. The present study addresses this issue by elucidating the mechanism governing the formation of Au NCs co-protected by both bidentate (SR′S) and monodentate (SR) ligands. The key impacts of ligand flexibility and site-specific exchange kinetics are identified, thereby providing crucial insights to support the strategic design and synthesis of stable, multi-site SR-protected Au NCs with rigid, well-defined architectures.

## Abstract 2

Multi-site ligands stabilize gold nanoclusters but often cause unwanted inter-cluster cross-linking. This study overcomes this by leveraging bidentate/monodentate ligand combinations and flexibility to synthesize stable, well-defined structures.

## Graphical abstract

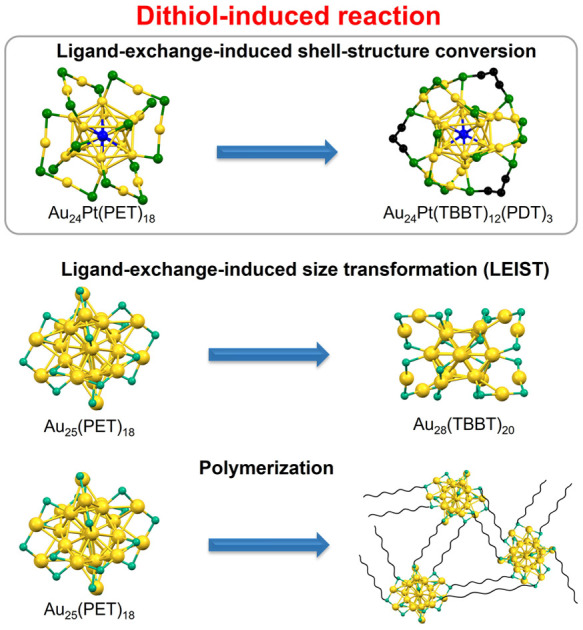

## Introduction

Ligand-protected metal nanoclusters (NCs) with atomically precise composition exhibit unique geometric and electronic structures distinct from those of the corresponding bulk metals or larger metal nanoparticles (NPs).^1–9^ These structural features endow them with exceptional properties and wide applicability in diverse areas, including catalysis, luminescence, and magnetic materials.^10–17^ Notably, the high-performance properties of such NCs are directly correlated with their precise atomic arrangements, which can be elucidated through single-crystal X-ray diffraction (SC-XRD) analysis and theoretical calculations. This structural clarity is crucial because it enables a fundamental understanding of structure–property relationships to support the rational design of next-generation metal NCs with enhanced functionality.^7,18^

For example, thiolate (SR)-protected gold (Au) NCs (*i.e.*, Au_*m*_(SR)_*n*_, where *m* denotes the number of Au atoms, and *n* represents the number of SR ligands) have high stability owing to the strong Au–S bonds; they have also shown high activity in various catalytic reactions.^19,20^ Lee *et al.* demonstrated that doping [Au_25_(SC_6_H_13_)_18_]^−^ (SC_6_H_13_ = 1-hexanethiolate) with a single equivalent of Pt to form [Au_24_Pt(SC_6_H_13_)_18_]^0^ significantly enhanced the NC's hydrogen evolution reaction (HER) activity, with approximately twice the reaction rate of commercial Pt NP catalysts.^21^ Our group conducted a comprehensive study investigating how various structural parameters of Au_*m*_(SR)_*n*_ NCs (*e.g.*, the number of constituent atoms, ligand chain length, and alloying) influenced their HER activity.^22^ On this basis, we designed a novel bimetallic NC, [Au_24_Pt(TBBT)_12_(PDT)_3_]^0^ (TBBT = 4-*tert*-butylbenzenethiolate; PDT = 1,3-propanedithiolate) by optimizing the structural features to promote the HER. The catalytic activity of [Au_24_Pt(TBBT)_12_(PDT)_3_]^0^ is approximately five-fold higher than that of its monodentate SR-protected analogue, [Au_24_Pt(SC_6_H_13_)_18_]^0^ and [Au_24_Pt(TBBT)_18_]^0^.^23^

**Fig. 1:**
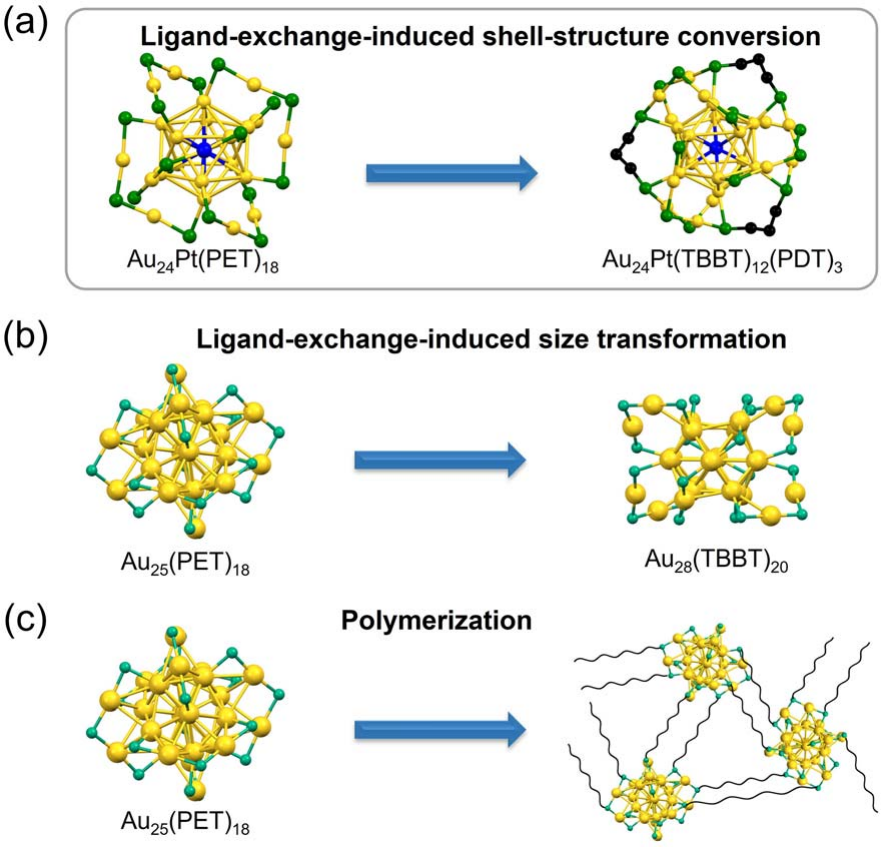
Reactions involving Au_*m*_(SR)_*n*_ with dithiolate: (a) this work, (b) ligand-exchange-induced size transformation, and (c) polymerization. For clarity, ligands not involved in bridging have been omitted. Color code: yellow = Au, green = S, blue = Pt, and black/gray = C.

In addition to NCs protected solely by monodentate SRs, Au NCs co-protected by monodentate SRs and bidentate SRs (SR′S)—denoted as Au_*m*_(SR)_*n*_(SR′S)_*l*_—can be synthesized *via* ligand-exchange reactions.^23,24^ Precise control over the reaction conditions, particularly temperature and the ligand molar ratio, allows for the isolation of Au_*m*_(SR)_*n*_(SR′S)_*l*_ NCs. Notably, the prepared [Au_24_Pt(SR)_12_(SR′S)_3_]^0^ retains a regular icosahedral Au_12_Pt core that is structurally analogous to that of prototypical Au_24_M(SR)_18_ NCs (Fig. 1).^25–27^ Additionally, [Au_24_Pt(SR)_12_(SR′S)_3_]^0^ has a significantly constrained surface structure due to the cross-linking of staple motifs by the SR′S ligands. This structural constraint exposes the metal core atoms to the external environment (Fig. 1(a)). Such accessible Au_12_M cores are expected to facilitate substrate access, thereby supporting catalytic activity for the HER, as well as a diverse range of other catalytic reactions.

**Fig. 2:**
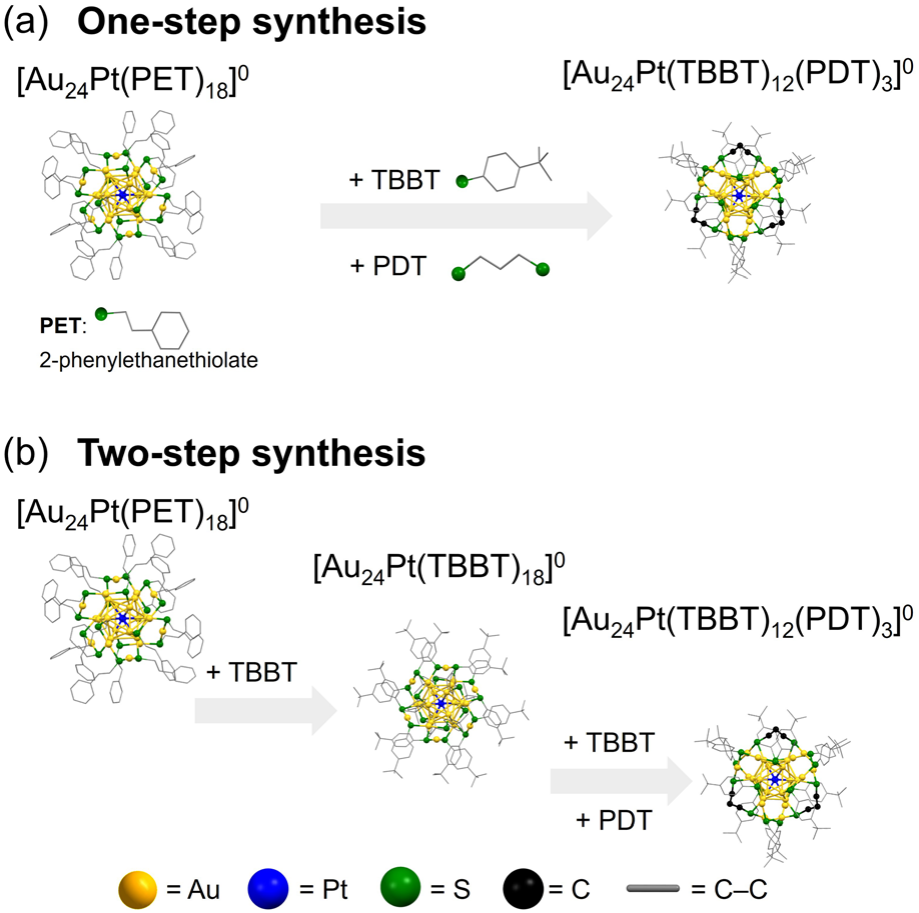
Schematic of the (a) one-step and (b) two-step synthetic methods for preparing [Au_24_Pt(TBBT)_12_(PDT)_3_]^0^ from [Au_24_Pt(PET)_18_]^0^.

However, the precise formation mechanism of [Au_24_Pt(SR)_12_(SR′S)_3_]^0^ remains elusive. This species is unique in that it is obtained exclusively through a shell (staple) structural transformation, which avoids the common pitfalls of size conversion or polymerization (Fig. 1(b, c) and 2). This study included a multi-faceted investigation into the synthesis of [Au_24_Pt(SR)_12_(SR′S)_3_]^0^ and explored strategies to increase the incorporation of SR′S ligands. The results indicate that the successful creation of Au NCs co-protected by SR and SR′S depends on two critical factors: (1) the selection of SR′S ligands with appropriate S–S bridging distances, and (2) the regulation of internal core Au–staple S distances by incorporating monodentate SRs with optimized binding affinities for the Au surface. These insights were implemented to synthesize novel [Au_24_Pt(SR)_10_(SR′S)_4_]^0^ NCs, which have a higher degree of SR′S incorporation than reported thiolate-protected NCs to date.^23^ These findings can inform the strategic design and synthesis of novel Au_*m*_(SR)_*n*_(SR′S)_*l*_ architectures with broad functional applicability in various fields.

## Results and discussion

### Influence of monothiolate on the synthesis of mono/dithiolate-co-protected Au NCs

[Au_11_(PPh_3_)_7_(X)_3_]^0^ (PPh_3_ = triphenylphosphine) is an established Au NC protected by monodentate phosphines (PR) that is relatively unstable.^28–30^ However, its stability can be enhanced significantly upon protection with bidentate phosphines (PR′P), which stabilizes the [Au_13_(DPPE)_5_X_2_]^3+^ (DPPE = 1,2-bis(diphenylphosphino)ethane) core with a regular icosahedral geometry (Fig. S1).^31,32^ Meanwhile, owing to the superior binding affinity of SR toward Au, various Au_*m*_(SR)_*n*_ NCs remain stable even when protected by monodentate SR ligands. Therefore, Au NCs protected by SR′S ligands are expected to exhibit high stability. Nevertheless, the synthesis of such Au_*m*_(SR′S)_*l*_ NCs have not yet been realized, likely because introducing SR′S ligands frequently causes ligand-exchange-induced size/structural transformation (LEIST),^33–38^ resulting in the formation of Au NCs with unintended nuclearities (Fig. 1(b)). Moreover, the strong Au–S bonds of SR′S promote inter-NC linkages, leading to polymerization (Fig. 1(c)). Recent studies have demonstrated that the use of SR′S ligands with a carbon chain length of six (*i.e.*, 1,6-hexanedithiol) causes the formation of disulfide bonds between sulfur termini, thereby linking Au_*m*_(SR)_*n*_ units.^39^ Consequently, to our knowledge, there are no reported examples of isolated Au_*m*_(SR′S)_*l*_ protected by solely SR′S ligands.

Recently, Au NCs co-protected by both monodentate and bidentate SRs have been reported, such as [Au_24_Pt(TBBT)_12_(SR′S)_3_]^0^ and [Au_28_(TBBT)_18_(SR′S)_1_]^0^.^23,24^ In the case of [Au_24_Pt(TBBT)_12_(SR′S)_3_]^0^, the combination of a rigid Au_12_Pt core and the presence of monodentate SR ligands suppresses LEISTs and prevents NC polymerization. Instead, this configuration limits the structural changes to only the transformation of staple (shell) motifs *via* SR′S-mediated cross-linking (Fig. 1(a)). Thus, it is crucial to elucidate the role of monodentate SRs in the synthesis of Au_*m*_(SR)_*n*_(SR′S)_*l*_ to comprehensively understand these synthetic design strategies.

Previous studies have shown that when [Au_24_Pt(PET)_18_]^0^ (PET = 2-phenylethanethiolate) serves as a precursor for ligand exchange with bulky TBBT, all ligands are substituted while leaving the underlying framework intact, resulting in the selective synthesis of [Au_24_Pt(TBBT)_18_]^0^ (Fig. 2(b)).^40^ This stability is particularly noteworthy given that the same reaction using [Au_25_(PET)_18_]^−^ leads to structural transformations into Au_*m*_(SR)_*n*_ NCs with varying nuclearities, such as Au_28_(TBBT)_20_, Au_23_(TBBT)_17_, and Au_20_(TBBT)_16_.^41–44^ This structural integrity is attributed to the inherently robust framework of the Au_12_Pt core in [Au_24_Pt(SR)_18_]^0^.

In this study, [Au_24_Pt(SR)_18_]^0^ was selected as a precursor for the introduction of SR′S ligands because it does not undergo unintended structural core rearrangements. Geometric investigations of the S–S distances in [Au_24_Pt(SR)_18_]^0^ suggested that SR′S ligands with a carbon chain length of two or three (*i.e.*, S(CH_2_)_*x*_S, where *x* = 2 or 3) are sterically appropriate for bridging adjacent SR′S ligands.^23^ However, the addition of 1,3-propanedithiol (PDTH_2_) to [Au_24_Pt(PET)_18_]^0^ resulted in the formation of insoluble materials, rather than the desired SR′S-protected NCs. This was likely due to (i) the high stability of the Au–S bonds in the PET ligands, which may inhibit the exchange process, or (ii) the onset of inter-NC polymerization induced by an excess of PDT (Fig. 1(c)).

In contrast, the reaction of [Au_24_Pt(TBBT)_18_]^0^ with PDTH_2_ readily yielded [Au_24_Pt(TBBT)_12_(PDT)_3_]^0^ (Fig. 2(b)). To better understand this reactivity, the structural differences between [Au_24_Pt(PET)_18_]^0^ and [Au_24_Pt(TBBT)_18_]^0^ were examined. Although these NCs shared essentially the same framework, they exhibited distinct structural distortions. Analysis of the average bond lengths (Fig. S2) revealed that the Au_surface_–S_surface_ (*i.e.*, the Au–S bond between the surface Au of the Au_12_Pt core and staple S) was longer in [Au_24_Pt(TBBT)_18_]^0^ than in [Au_24_Pt(PET)_18_]^0^. Consequently, [Au_24_Pt(TBBT)_18_]^0^ had a more compact Au_12_Pt core, with the Au_2_(SR)_3_ staples positioned further from the core surface than in [Au_24_Pt(PET)_18_]^0^. The difference in the p*K*_a_ values of the aromatic TBBT and aliphatic PET ligands suggested that TBBT has a lower electron-donating ability of the S atoms.^40^ This leads to weaker Au–S bonds in [Au_24_Pt(TBBT)_18_]^0^, which facilitate the displacement of Au_2_(SR)_3_ staples from the metal core. These structural and electronic factors account for the differing chemical and thermodynamic stabilities of the two NCs, with [Au_24_Pt(TBBT)_18_]^0^ exhibiting lower stability. Indeed, [Au_24_Pt(TBBT)_18_]^0^ can only be obtained *via* ligand exchange and cannot be synthesized through conventional co-reduction methods. These results indicate that ligand exchange with SR′S ligands is promoted by (i) maintaining an appropriate S–S distance within the NCs and (ii) using a monodentate SR ligand with a relatively weak Au–S bond to facilitate the exchange process (Fig. 2).

Furthermore, the reaction proceeded when using 4-isopropylbenzenethiolate (IPBT), an aromatic ligand similar to TBBT, to form [Au_24_Pt(IPBT)_12_(PDT)_3_]^0^ (Fig. S3). In contrast, the reaction barely proceeded when using SC_6_H_13_,^23^ an aliphatic ligand similar to PET. These observations suggest that aromatic ligands (*e.g.*, TBBT and IPBT), which have weaker Au–S bonds than those of aliphatic ligands (*e.g.*, PET, SC_6_H_13_, and SC_12_H_25_ [1-dodecanethiolate]), are more readily substituted by PDT ligands.

### Reaction tracking from [Au_24_Pt(TBBT)_18_]^0^ or [Au_24_Pt(PET)_18_]^0^ to [Au_24_Pt(TBBT)_12_(PDT)_3_]^0^

**Fig. 3:**
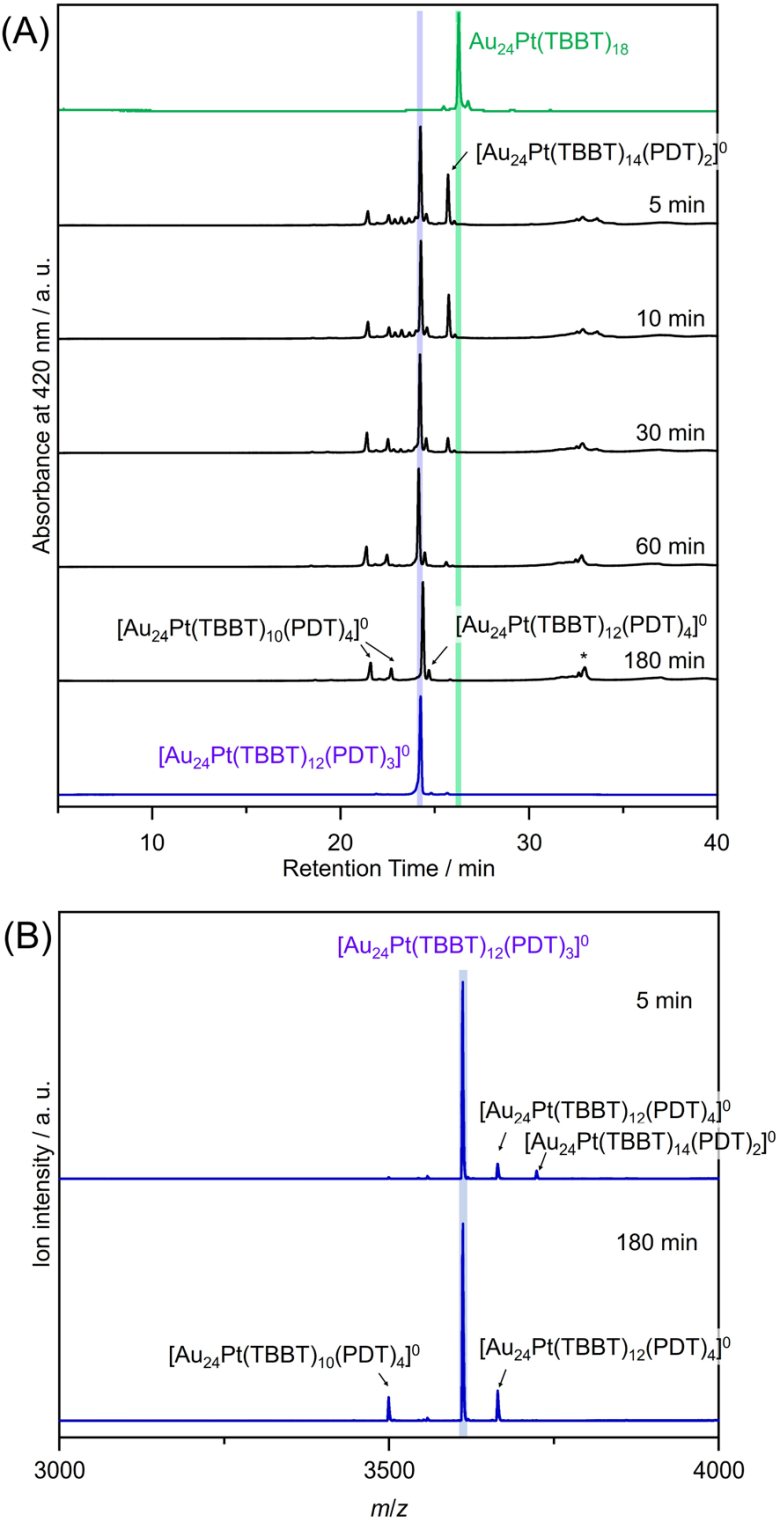
(A) Reverse-phase high-performance liquid chromatograms at 0, 5, 10, 30, 60, and 180 min; (B) negative-ion electrospray-ionization mass spectra of products obtained from the reaction of [Au_24_Pt(TBBT)_18_]^0^ with TBBTH and PDTH_2_ for 5 and 180 min. [Au_24_Pt(TBBT)_10_(PDT)_4_]^0^ exhibited several structural isomers with differing retention times. The peak at a retention time of approximately 32 min (*) likely represents an aggregate of multiple NCs, but its intensity was insufficient for MS detection.

**Fig. 4:**
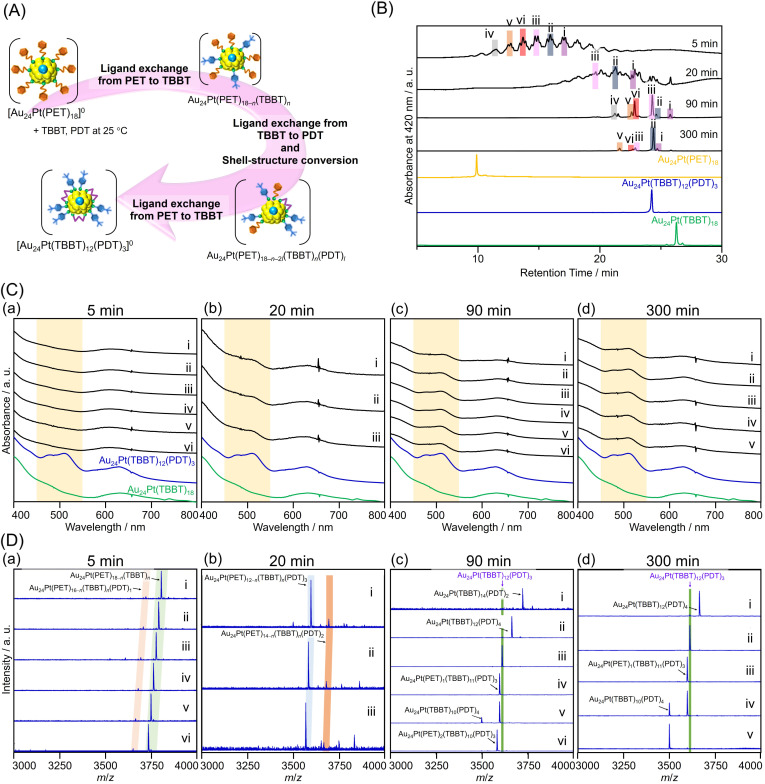
(A) Schematic illustration of the transformation from [Au_24_Pt(PET)_18_]^0^ to [Au_24_Pt(TBBT)_12_(PDT)_3_]^0^; (B) RP-HPLC chromatograms; (C) UV-vis optical absorption spectra obtained with a PDA detector attached to the HPLC apparatus; (D) negative-ion ESI mass spectra of products obtained following the reaction of [Au_24_Pt(PET)_18_]^0^ with TBBTH and PDTH_2_ for (a) 5, (b) 20, (c) 90, and (d) 300 min. Each peak was mainly attributed as follows. At 5 min: (i–vi) Au_24_Pt(PET)_18−*n*_(TBBT)_*n*_ (*n* = 3–8); at 20 min: (i) Au_24_Pt(PET)_1_(TBBT)_11_(PDT)_3_, (ii) Au_24_Pt(PET)_2_(TBBT)_10_(PDT)_3_, (iii) Au_24_Pt(PET)_3_(TBBT)_9_(PDT)_3_; at 90 min: (i) Au_24_Pt(TBBT)_14_(PDT)_2_, (ii) Au_24_Pt(TBBT)_12_(PDT)_4_, (iii) Au_24_Pt(TBBT)_12_(PDT)_3_, (iv and v) Au_24_Pt(PET)_1_(TBBT)_11_(PDT)_3_, (vi) Au_24_Pt(PET)_2_(TBBT)_10_(PDT)_3_; at 300 min: (i) Au_24_Pt(TBBT)_12_(PDT)_4_ (ii–iv) Au_24_Pt(PET)_12−*n*_(TBBT)_*n*_(PDT)_3_, (v) Au_24_Pt(TBBT)_10_(PDT)_4_.

Our group previously established a methodology for the atomically precise separation of various SR-protected NCs using reverse-phase high-performance liquid chromatography (RP-HPLC).^45–47^ This separation is primarily driven by the charge state, ligand types, and alloy configurations of the NCs. It is also possible to characterize the electronic structures of Au_*m*_(SR)_*n*_ by using a photodiode array (PDA) detector integrated within the HPLC system (Fig. S4). These methods were applied to investigate the chemical composition, elemental distribution, and electronic structures of the prepared materials by coupling RP-HPLC with electrospray-ionization mass spectrometry (ESI-MS).^48,49^ Previous research on this reaction system demonstrated that for [Au_24_Pt(PET)_18_]^0^, a ligand-exchange reaction at 25 °C with a [TBBTH] : [PET] ratio of approximately 250 : 1 over more than 10 hours yielded [Au_24_Pt(TBBT)_18_]^0^.^42^ The present study aimed to prepare [Au_24_Pt(TBBT)_12_(PDT)_3_]^0^*via* ligand-exchange synthesis with a [PDTH_2_] : [TBBTH] : [PET] ratio of 12 : 239 : 1 at 25 °C. Fig. 3 and 4 show chromatograms highlighting the retention times of the precursor [Au_24_Pt(PET)_18_]^0^ (10.0 min), the fully-TBBT-exchanged [Au_24_Pt(TBBT)_18_]^0^ (27.0 min), and the target product [Au_24_Pt(TBBT)_12_(PDT)_3_]^0^ (24.2 min). The appearance of a single peak in each chromatogram confirms that these NCs were obtained with high purity.

The shift in retention times can be explained by the surface polarity of the NCs. The TBBT ligands have lower polarity than the precursor PET ligands owing to their structural symmetry and the steric bulk of the *tert*-butyl groups surrounding the sulfur atoms. In reverse-phase separation mode, higher polarity species elute earlier (*i.e.*, with shorter retention times), whereas lower polarity (more hydrophobic) species are retained longer (Fig. S4). Thus, the systematic increase in the number of TBBT ligands resulted in a corresponding shift toward longer retention times in the experimental data.

#### Reaction tracking from [Au_24_Pt(TBBT)_18_]^0^ to [Au_24_Pt(TBBT)_12_(PDT)_3_]^0^

The results of the ligand-exchange reaction of [Au_24_Pt(TBBT)_18_]^0^ with PDT (Fig. 2(b)) are presented in Fig. 3. The reaction proceeded rapidly; within 5 minutes, the primary product was identified as Au_24_Pt(TBBT)_12_(PDT)_3_ (Fig. 3A). Qualitative analysis revealed that a negligible amount of Au_24_Pt(TBBT)_16_(PDT)_1_ was formed, while Au_24_Pt(TBBT)_14_(PDT)_2_ and Au_24_Pt(TBBT)_10_(PDT)_4_ were obtained in higher yields as byproducts (Fig. 3B). The scarcity of the mono-substituted Au_24_Pt(TBBT)_16_(PDT)_1_ suggested that initial substitution occurred at a core site where the S–S distance in the Au_24_Pt(TBBT)_18_ framework is appropriate for SR′S coordination. Owing to the relative flexibility of the staple structure in solution, the PDT ligand could rapidly bind to a thermodynamically stable core site. The subsequent introduction of a second PDT ligand to form Au_24_Pt(TBBT)_14_(PDT)_2_ likely triggered a shell structural transformation toward a staple configuration similar to that of the stable Au_24_Pt(TBBT)_12_(PDT)_3_.

The structural flexibility of PDT is critical for these transformations. It is reasonable to presume that PDT coordinated to Au_24_Pt(TBBT)_12_(PDT)_3_ in a folded conformation, which facilitated the cross-linking required to form Au_24_Pt(TBBT)_10_(PDT)_4_. The importance of backbone flexibility and length is further supported by comparative experiments. The addition of HS(CH_2_)_2_SH to Au_24_Pt(TBBT)_12_(PDT)_3_ yielded no reaction, whereas the use of HS(CH_2_)_4_SH generated a 4-SR′S-substituted product (refer to “Synthesis of other dithiolate-protected Au NCs” section). Additionally, the lack of a reaction between rigid 1,3-benzenedithiol (BDTH_2_) and Au_24_Pt(TBBT)_18_ indicated that a specific flexible chain length is essential for effective SR′S cross-linking.^23^

After 180 minutes of reaction, the peak corresponding to Au_24_Pt(TBBT)_14_(PDT)_2_ disappeared, and peaks attributable to the Au_24_Pt(TBBT)_10_(PDT)_4_ and Au_24_Pt(TBBT)_12_(PDT)_4_ byproducts were observed (Fig. 3). The formation of Au_24_Pt(TBBT)_12_(PDT)_4_ was attributed to trace amounts of (PDTH)_2_, which is a disulfide-bonded impurity present in the PDTH_2_ precursor. This (PDTH)_2_ species could induce inter-NC cross-linking, rather than the intra-NC bridging observed with monomeric PDT.

#### Reaction tracking from [Au_24_Pt(PET)_18_]^0^ to [Au_24_Pt(TBBT)_12_(PDT)_3_]^0^

[Au_24_Pt(TBBT)_12_(PDT)_3_]^0^ can also be synthesized *via* a one-step ligand-exchange method following the simultaneous addition of TBBTH and PDTH_2_ to the [Au_24_Pt(PET)_18_]^0^ precursor (Fig. 2(a)). This approach provides a convenient route to obtain the target [Au_24_Pt(TBBT)_12_(PDT)_3_]^0^ in relatively high yield. To better understand this synthetic process, the reaction mechanism governing this one-step process was investigated (Fig. 4).

Fig. 4B–D present the RP-HPLC, UV-vis, and ESI-MS results over time after the simultaneous addition of PDTH_2_ and TBBTH to [Au_24_Pt(PET)_18_]^0^. After 5 minutes (Fig. 4(a)), the RP-HPLC chromatogram (Fig. 4B) contained multiple peaks at retention times longer than that of the precursor [Au_24_Pt(PET)_18_]^0^. These peaks were interpreted as intermediate species formed during the ligand-exchange of PET with TBBT. At this stage, the optical absorption spectra lacked the characteristic features of [Au_24_Pt(SR)_12_(SR′S)_3_]^0^ that are typically observed in the 450–550 nm range (Fig. 4C(a)). This suggests that the initial phase of the reaction was dominated by PET-to-TBBT ligand exchange, with negligible PDT-mediated cross-linking. This was further supported by ESI-MS analysis of the representative peaks (Fig. 4D(a); i–vi), which were assigned to Au_24_Pt(PET)_18−*n*_(TBBT)_*n*_ (*n* = 3–8). These results confirmed that there was negligible PDT substitution during the early stages of the reaction. Previous studies examining the ligand-exchange reaction between [Au_25_(PET)_18_]^−^ and PDTH_2_ indicated that the process typically yielded a polydisperse mixture of Au_25_(PET)_18−2*l*_(PDT)_*l*_ (*l* = 2–6), rather than a discrete product.^23,50^ In the present study, the molar ratio of PDT to TBBT was low ([PDTH_2_] : [TBBTH] ratio of 1 : 20), and as a result, PET–PDT exchange products such as Au_24_Pt(PET)_18−2*l*_(PDT)_*l*_ were scarcely detected. These observations support the hypothesis that PDT substitution occurs preferentially at Au–TBBT sites, rather than Au–PET sites, consistent with the mechanistic behavior discussed above.

Fig. 4(b) shows the results of the reaction after 20 minutes. The optical absorption spectra of the peaks with longer retention times in Fig. 4B reveal a broadening and splitting of the features in the 450–550 nm region (Fig. 4C(b)). This spectral evolution was primarily attributed to the emergence of the characteristic peaks of [Au_24_Pt(SR)_12_(SR′S)_3_]^0^, which originate from the staple structures cross-linked by PDT. ESI-MS analysis of peaks (i–iii) identified the species as Au_24_Pt(PET)_1_(TBBT)_11_(PDT)_3_, Au_24_Pt(PET)_2_(TBBT)_10_(PDT)_3_, and Au_24_Pt(PET)_3_(TBBT)_9_(PDT)_3_, respectively (Fig. 4D(b)). Based on these observations, it is presumed that subsequent ligand substitution to form PDT_*l*_ (*l* ≤ 3) proceeds once the (PET)_2*n*_ (*n* ≤ 3) core sites with appropriate S–S distances are replaced by TBBT. Our group previously demonstrated that during the ligand exchange of SC_12_H_25_ with [Au_24_Pd(PET)_18_]^0^, the SC_12_H_25_ ligands first preferentially replaced the PET ligand at the core site, yielding the monosubstituted isomer [Au_24_Pd(PET)_17_(SC_12_H_25_)]^0^.^48^ Thus, in the present study, the following three-step mechanism is proposed. First, the chain-like PDTH_2_ coordinates to the core-site TBBT as an S–(CH_2_)_3_–SH group. This is followed by rapid replacement by TBBT ligands that coordinate to the core at sites with optimal S–S distances. Finally, the conversion of the staple structure occurs nearly simultaneously with these exchange events. This mechanism is consistent with the experimental observation that both Au_24_Pt(PET)_16−*n*_(TBBT)_*n*_(PDT)_1_ (13 ≥ *n* ≥ 4) and Au_24_Pt(PET)_12−*n*_(TBBT)_*n*_(PDT)_3_ (*n* = 3, 5, 6) were present as early as 5 minutes after reaction initiation.

Furthermore, after 90 minutes of reaction (Fig. 4(c)), the optical absorption spectrum exhibited complete splitting of the peak in the 450–550 nm region, suggesting that the conversion to the cross-linked staple structure was nearly complete following PDT introduction (Fig. 4C(c)). Peaks were assigned *via* ESI-MS analysis (Fig. 4D(c)) as Au_24_Pt(TBBT)_14_(PDT)_2_ (i), Au_24_Pt(TBBT)_12_(PDT)_4_ (ii), Au_24_Pt(TBBT)_10_(PDT)_4_ (v), and various Au_24_Pt(PET)_12−*n*_(TBBT)_*n*_(PDT)_3_ intermediates (iii, iv and vi). These results indicated that after 90 minutes, the system had largely transitioned to thermodynamically stable cross-linked configurations, although some residual PET ligands remained within the TBBT/PDT framework.

The results after 300 minutes of reaction are presented in Fig. 4(d). Peaks (i–v) were observed at four distinct retention times (Fig. 4B). All corresponding optical absorption spectra showed complete splitting of the peak around 450–550 nm, consistent with the observations after 90 minutes (Fig. 4C(d)). ESI-MS assignments confirmed the formation of Au_24_Pt(PET)_12−*n*_(TBBT)_*n*_(PDT)_3_ (ii–iv) and Au_24_Pt(TBBT)_*n*_(PDT)_4_ (where *n* = 10 or 12; i, and v) (Fig. 4D(d)). However, in contrast to the 90-minute samples, Au_24_Pt(TBBT)_14_(PDT)_2_ was no longer detected. This suggested that as the reaction approached equilibrium, the intermediate products converged to the target Au_24_Pt(TBBT)_12_(PDT)_3_.

**Fig. 5:**
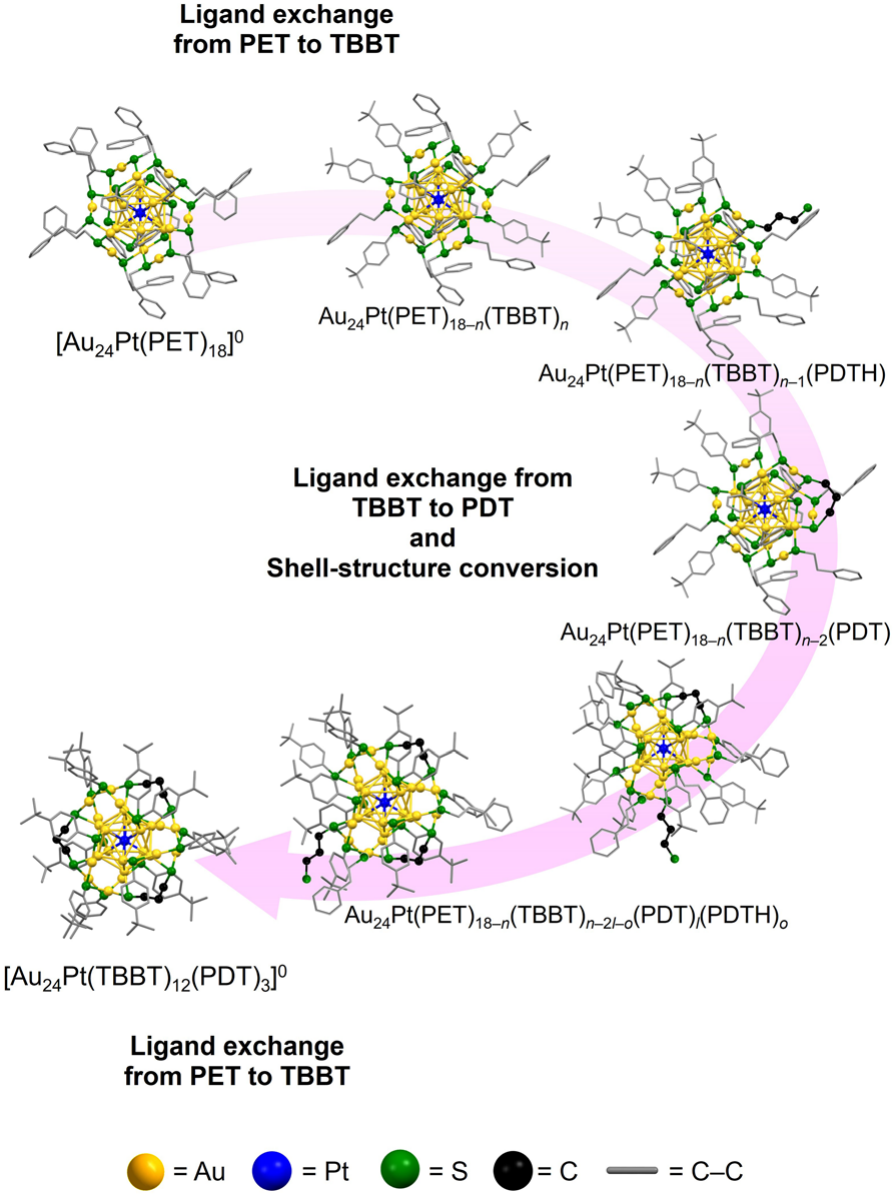
Schematic illustration of the detailed transformation mechanism from [Au_24_Pt(PET)_18_]^0^ to [Au_24_Pt(TBBT)_12_(PDT)_3_]^0^.

Based on the comprehensive analysis of the experimental data, the following mechanism is proposed for the ligand exchange of [Au_24_Pt(PET)_18_]^0^ using PDT and TBBT to obtain Au_24_Pt(TBBT)_12_(PDT)_3_ (Fig. 4A and 5). (1) Initial ligand exchange: the reaction is initiated with the preferential exchange of PET ligands for TBBT. (2) PDT coordination: ligand exchange occurs between the newly substituted TBBT and PDTH at the core-site S atom. (3) Staple conversion: rapid ligand exchange occurs between TBBT—which provides a sterically appropriate S–S distance—and the opposite terminal S of the already-ligated PDTH; this process facilitates the conversion of the shell structure to the cross-linked staple configuration. (4) Final substitution: all remaining PET ligands undergo exchange with TBBT, resulting in the stable [Au_24_Pt(TBBT)_12_(PDT)_3_]^0^ (Fig. 4A and 5). This proposed mechanism was further validated by observing the same behavior during the ligand exchange from [Au_24_Pt(SC_6_H_13_)_18_]^0^ to [Au_24_Pt(TBBT)_12_(PDT)_3_]^0^ (Fig. S6–S11), suggesting that the preference for TBBT-mediated core substitution and subsequent PDT cross-linking are key characteristics supporting the formation of [Au_24_Pt(TBBT)_12_(PDT)_3_]^0^. [Au_24_Pt(TBBT)_12_(PDT)_3_]^0^ obtained *via* this reaction mechanism was confirmed to possess high thermal stability (Fig. S12).

### Synthesis of other dithiolate-protected Au NCs

Based on the findings discussed above, the syntheses of novel Au_*m*_(SR)_*n*_(SR′S)_*l*_ NCs were further explored. First, PDT was introduced as an SR′S ligand to determine the possibility of achieving higher substitution numbers. Structurally, [Au_24_Pt(TBBT)_12_(PDT)_3_]^0^ has three additional core sites available for further SR′S substitution (Fig. S13 and S14). If all available core sites are occupied, [Au_24_Pt(TBBT)_18−2*l*_(PDT)_*l*_]^0^ (*l* = 4–6) can form *via* a similar reaction pathway. However, although [Au_24_Pt(TBBT)_10_(PDT)_4_]^0^ formed relatively easily, it could not be isolated.

Additionally, the effect of the SR′S ligand alkyl chain length was investigated. When TBBTH and HS(CH_2_)_*x*_SH (*x* = 2, 4, 6) were reacted with [Au_24_Pt(PET)_18_]^0^, the process yielded insoluble precipitates, and no SR′S-protected NCs were obtained. Additionally, SR′S cross-linking was attempted using BDTH_2_, which has the same carbon chain length as PDTH_2_ but features a rigid benzene ring. This also failed to yield SR′S-protected NCs. The S–S distances bridged by PDT in [Au_24_Pt(TBBT)_12_(PDT)_3_]^0^ were similar (4.443, 4.497, and 4.497 Å) (Fig. S13). Thus, it is reasonable to conclude that [Au_24_Pt(TBBT)_12_(PDT)_3_]^0^ exhibits high stability due to the presence of these three core sites with S–S distances that are optimally suited for PDT coordination.

**Fig. 6:**
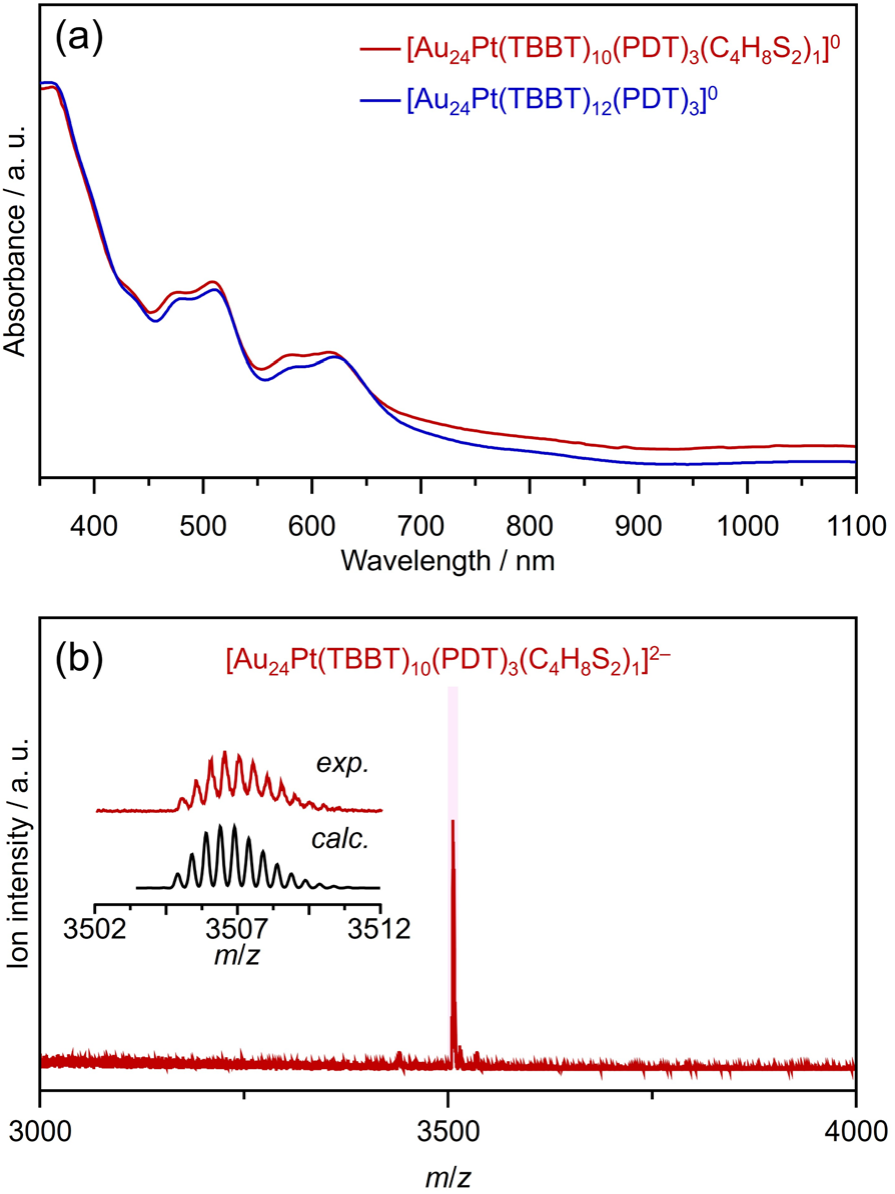
(a) UV-vis spectrum and (b) negative-ion ESI mass spectrum of 1. In (a), the absorbance spectra of Au_24_Pt(TBBT)_12_(PDT)_3_ are also shown for comparison.

Considering these findings, we posited that introducing additional SR′S ligands into [Au_24_Pt(TBBT)_12_(PDT)_3_]^0^ would require SR′S ligands with an S–S distance more compatible than that of PDT. To evaluate Au_*m*_(SR)_*n*_(SR′S)_*l*_ with a higher degree of substitution, [Au_24_Pt(TBBT)_12_(PDT)_3_]^0^ NCs were prepared with SR′S ligands of varying chain lengths. The addition of 1,2-ethanedithiol (HS(CH_2_)_*x*_SH; *x* = 2) to [Au_24_Pt(TBBT)_12_(PDT)_3_]^0^ did not initiate a reaction; however, the use of 1,4-butanedithiol (HS(CH_2_)_*x*_SH; *x* = 4) yielded a relatively stable product 1 that could be isolated (Fig. 6(a)). ESI-MS analysis identified the resulting product as [Au_24_Pt(TBBT)_10_(PDT)_3_(S(CH_2_)_4_S)_1_]^0^, in which two additional TBBT units were replaced by S(CH_2_)_4_S ligands (Fig. 6(b)). These results also suggest that both the carbon chain length and the inherent flexibility of the SR′S ligand are critical factors for increasing the number of SR′S ligands on the NC surface.

## Conclusions

This study provided the following key insights regarding the transformation of [Au_24_Pt(PET)_18_]^0^ into [Au_24_Pt(TBBT)_12_(PDT)_3_]^0^:

(1) Direct substitution of PET with PDT ligands is hindered by the strong Au–PET bonds, ultimately rendering the formation of [Au_24_Pt(PET)_12_(PDT)_3_]^0^ unfavorable.

(2) Aromatic ligands (*e.g.*, TBBT and IPBT), which have weaker Au–S bonds than aliphatic ligands (*e.g.*, PET, SC_6_H_13_, SC_12_H_25_), readily undergo substitution with the aliphatic PDT ligand.

(3) In the intermediate species Au_24_Pt(PET)_18−*n*_(TBBT)_*n*_, PDT facilitates intra-NC bridging at S sites with appropriate S–S distances, simultaneously inducing a transformation of the staple structure.

(4) PDT ligands that fail to support intra-NC bridging tend to induce inter-NC cross-linking, leading to polymerization.

These synthetic insights were implemented to synthesize [Au_24_Pt(TBBT)_10_(PDT)_3_(S(CH_2_)_4_S)_1_]^0^, which incorporates more SR′S ligands than in any previously reported material.^23^ It would be useful to estimate these substitution sites using theoretical calculations in the future.^51^

Indeed, Au_25_(SR)_18_ is recognized as a stable framework that can further incorporate various heterometals (*e.g.*, Ir, Rh, Pt, Pd, Ag, Cu, Hg, or Cd); however, excessive or highly dissimilar doping often compromises the material's geometric and electronic stability. The Au_*m*_(SR)_*n*_(SR′S)_*l*_ synthesized in this work have a rigid staple structure, which is expected to mitigate the instabilities typically associated with NC alloying. Consequently, the novel M_*y*_Au_25−*y*_(SR)_12_(SR′S)_3_ template can enable the development of NCs with enhanced functionality. This research provides valuable insights to support the design of novel metal NCs and their alloyed derivatives featuring rigid, cross-linked staple architectures.^52,53^

## Experimental

### Synthesis of 1

In the optimized synthesis, 8.6 mg [Au_24_Pt(TBBT)_12_(PDT)_3_]^0^ was dissolved in 4.0 mL toluene, to which 863 µL TBBT (98%) and 51.3 µL HS(CH_2_)_4_SH were added at room temperature, and the mixture was stirred for 3 hours to allow the ligand-exchange reaction to proceed. The product was washed sequentially with ultrapure water and a mixture of ultrapure water and methanol and then extracted with dichloromethane. Finally, the products were separated by PTLC (dichloromethane : hexane = 4 : 6), and the second layer was scraped off and extracted with dichloromethane to obtain [Au_24_Pt(TBBT)_10_(PDT)_3_(S(CH_2_)_4_S)_1_]^0^.

## Author contributions

T. Kawawaki and Y. Negishi designed the experiments and conducted the measurements with S. Yoshikawa and S. Hossain. T. Kawawaki, S. Hossain and Y. Negishi wrote the paper. All authors approved the final version of the manuscript.

## Conflicts of interest

There are no conflicts to declare.

## Supplementary Material

SC-017-D6SC00460A-s001

## Data Availability

Relevant data are available from the corresponding authors (T. Kawawaki and Y. Negishi) upon reasonable request. All data supporting the findings of this work are provided in the supplementary information (SI). Supplementary information: additonal experimental details, characterization and figures. See DOI: https://doi.org/10.1039/d6sc00460a.
