# Supplementary material for: Mechanism governing the formation of atomically precise dithiolate-protected gold nanoclusters
Source: Chem Sci. 2026 Mar 13;17(14):6931–8. doi: 10.1039/d6sc00460a (PMC13003514; doi:10.1039/d6sc00460a)
Supplement: SC-017-D6SC00460A-s001 [file SC-017-D6SC00460A-s001.pdf]

Supplementary Information (SI) for Chemical Science.
This journal is © The Royal Society of Chemistry 2026

Supporting Information

Mechanism Governing the Formation of Atomically Precise Dithiolate-Protected Gold Nanoclusters

Author Information

Sara Yoshikawa,[1,2,†] Tokuhisa Kawawaki,[1,2,†,*] Sakiat Hossain,[1,‡] and Yuichi Negishi[1,2,*]

Affiliations

[1] Carbon Value Research Center, Research Institute for Science and Technology, Tokyo University of Science, Kagurazaka, Shinjuku-ku, Tokyo 162–8601, Japan

[2] Institute of Multidisciplinary Research for Advanced Materials, Tohoku University, Katahira 2-1-1, Aoba-ku, Sendai 980-8577, Japan

[†] These authors contributed equally to this work.

*Corresponding author. E-mail: tokuhisa.kawawaki.d8@tohoku.ac.jp (T.K.), yuichi.negishi.a8@tohoku.ac.jp (Y.N.)

S1. Experimental Details

S1.1. Chemicals

All chemicals were obtained commercially and used as received. Silver nitrate, triphenylphosphine, dimethylsulfide, tetrahydrofuran (THF), methanol, toluene, hexane, dichloromethane, diethyl ether, acetonitrile, acetone, ethanol, *n*-pentane, and hydrogen peroxide were from Kanto Chemical Co., Inc. 4-*Tert*-butylbenzenethiol (TBBTH) (98%) and silica gel 60N, spherical neutral, particle size 63-210 μm were from FUJIFILM Wako Pure Chemical Co. Sodium tetrahydroborate ($NaBH_4$), tetrakis(triphenylphosphine)platinum(0) ($Pt(PPh_3)_4$), triethylamine, 1,3-propanedithiol ($PDTH_2$), 1,4-butanedithiol ($HS(CH_2)_4SH$), 1-hexanethiol, 4-isopropylbenzenethiolate (IPBT) and tetra-*n*-octylammonium bromide were from Tokyo Chemical Industry Co. 2-Phenylethanethiol (PETH) was from Sigma Aldrich. Hydrogen tetrachloroaurate(III) ($HAuCl_4$) tetrahydrate and hydrogen hexachloroplatinate(IV) (H_2PtCl_6) hexahydrate were from Tanaka Kikinzoku Kogyo. Alumina N 32-63 was from MP Biomedicals. Pure Milli-Q water (H_2O) (18.2 MΩ·cm) was generated using a Merck Millipore Direct 3 UV system.

S1.2. Synthesis

S1.2.1 Synthesis of $[Au_{24}Pt(PET)_{18}]^0$, $[Au_{24}Pt(TBBT)_{18}]^0$, and $[Au_{24}Pt(TBBT)_{12}(PDT)_3]^0$

$[Au_{24}Pt(PET)_{18}]^0$, $[Au_{24}Pt(TBBT)_{18}]^0$, and $[Au_{24}Pt(TBBT)_{12}(PDT)_3]^0$ were synthesized according to literature procedures.[1]

S1.2.2 Synthesis of $[Au_{24}Pt(SC_6H_{13})_{18}]^0$

$[Au_{24}Pt(SC_6H_{13})_{18}]^0$ was synthesized using a slightly modified reported method.[2] First, 1.60 mmol $HAuCl_4$, 0.4 mmol H_2PtCl_6, 4.64 mol tetra-*n*-octylammonium bromide were dissolved in 120 mL THF, and the resulting solution was stirred at room temperature for 45 minutes. Next, 13.4 mmol 1-hexanethiol was added to the

[‡] This author is currently affiliated with the Department of Chemical Engineering, Queen's University, Kingston, Ontario K7L 3N6, Canada

solution and stirred at room temperature for an additional 45 minutes. Subsequently, 20 mL of pure water cooled to 0 °C was added. After 2-3 minutes, a solution of 40 mmol sodium tetrahydroborate dissolved in 10 mL pure water cooled to 0 °C was added rapidly. After stirring the solution for 5 hours, the solvent was evaporated using a rotary evaporator, and the product was washed gradually from water to methanol. The precipitate was washed and purified on a silica column (toluene:hexane = 1:5). The solvent was evaporated from the elution, and the solid product was added to a mixture of 5 mL hydrogen peroxide solution and 5 mL dichloromethane and stirred for 5 hours. The product was then washed with pure water and purified by thin-layer chromatography (TLC; dichloromethane:hexane = 1:5) to obtain highly pure $[Au_{24}Pt(SC_6H_{13})_{18}]^0$.

S1.2.3 Synthesis of $[Au_{24}Pt(IPBT)_{12}(PDT)_3]^0$

In the optimized synthesis, 5.0 mg $[Au_{24}Pt(PET)_{18}]^0$ was dissolved in 3.4 mL toluene, to which 441 μL IPBT (94%) and 14.5 μL of $PDTH_2$ were added at 60 °C, and the mixture was stirred for 1.5 hours to allow the ligand-exchange reaction to proceed. The product was washed sequentially with ultrapure water and a mixture of ultrapure water and methanol and then extracted with dichloromethane. Finally, the products were separated by PTLC (dichloromethane:hexane = 7:13), and the top layer was scraped off and extracted with dichloromethane to obtain $[Au_{24}Pt(IPBT)_{12}(PDT)_3]^0$.

S1.3. Characterization

The diffuse reflection spectra were acquired at ambient temperature with a V-670 spectrometer (JASCO, Tokyo, Japan). The wavelength-dependent optical data [$I(w)$] were converted to energy-dependent data [I(E)] according to the following equation that conserved the integrated spectral areas: $I(E) = I(w)/|\partial E/\partial w| \propto I(w) \times w^2$.
ESI-MS measurements were performed with a microTOF II reflectron time-of-flight mass spectrometer (Bruker, Massachusetts, USA). In these measurements, an NC solution with a concentration of ~10 μg/mL in a mixture of dichloromethane and methanol was electrosprayed at a flow rate of 180 μL/h.

S1.4. Separation and isolation of product obtained by ligand-exchange reaction[3]

A Shimadzu Prominence high-performance liquid chromatography (HPLC) system comprising 2 LC-20AD pumps, an SPD-20A photodiode array (PDA) detector, a CTO-20A column oven, and a DGU-20A on-line degasser was used to separate and isolate the products. A YMC core-shell ODS column (Meteoric Core C18, 150 mm × 4.6 mm, inner diameter = 5 μm) was used as the stationary phase. The column and detector were aged (stabilized) for sufficient time before analysis. Reverse-phase (RP) mode was used for the separation. In the experiments, each precursor NC or the product obtained by ligand exchange was first suspended in a mixture of diethyl ether and acetonitrile (7:13). Then, 20 μL of the mixture was injected into a selecting valve. The mobile phase was continuously changed from pure acetonitrile to pure diethyl ether according to the gradient program shown in Fig. S4. The flow rate of the mobile phase was 1.0 mL/min, and the optical absorption spectra of the separated products were obtained using the PDA detector in the range of 190-800 nm. The chromatograms were obtained from the absorption intensity at 420 nm.

S2. Supplementary Figures

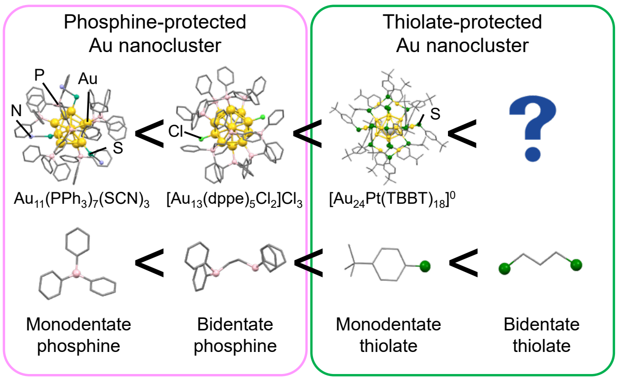

Fig. S1 Relationship between ligand coordination number and the geometric structure and thermodynamic stability of Au NCs.[4–6] Bidentate phosphines enhance the stability of Au NCs relative to monodentate phosphines. Meanwhile, thiolate ligands support high stability NCs, even as monodentate ligands. Au NCs protected by bidentate thiolates are expected to exhibit high stability.

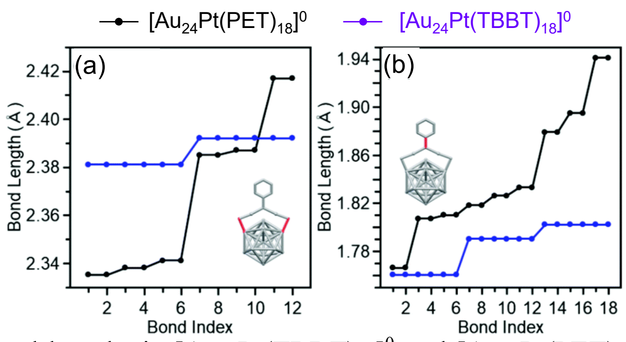

Fig. S2 Comparison of bond lengths in $[Au_{24}Pt(TBBT)_{18}]^0$ and $[Au_{24}Pt(PET)_{18}]^0$: (a) $Au_{surface}$–$S_{surface}$ and (b) S–C bonds.[6] Reproduced from Ref. 6 with permission from the Royal Society of Chemistry.

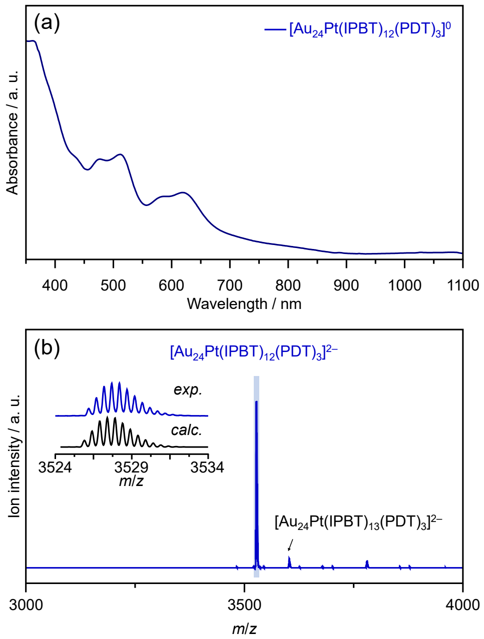

Fig. S3 (a) UV-vis optical absorption spectrum and (b) negative-ion ESI mass spectrum of $[Au_{24}Pt(IPBT)_{12}(PDT)_3]^0$

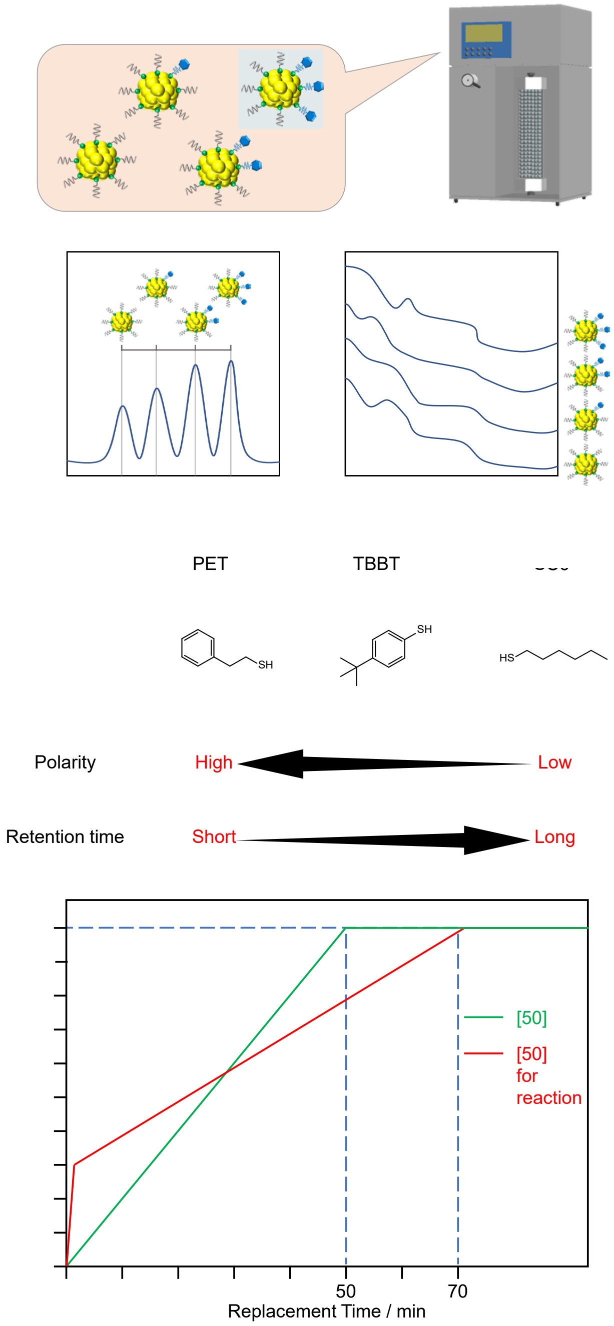

Fig. S4 (a) Detection of intermediates in ligand-exchange reactions involving metal NCs using HPLC; (b) relationship between ligand structure and polarity and HPLC detection time; (c) gradient program used for substitution of the mobile phase. The mobile phase was generally substituted from 100 vol.% of the adsorption solvent (acetonitrile) to 100 vol.% of the elution solvent (diethyl ether) within 50 minutes.

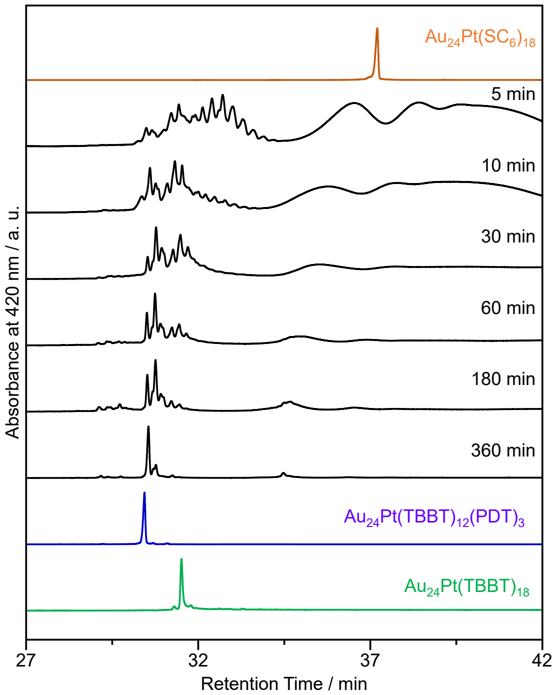

Fig. S5 RP-HPLC chromatograms of products obtained following the reaction between $[Au_{24}Pt(SC_6H_{13})_{18}]^0$, $PDTH_2$, and TBBTH for 5, 10, 30, 60, 180, and 360 minutes. RP-HPLC chromatograms of $[Au_{24}Pt(SC_6H_{13})_{18}]^0$, $[Au_{24}Pt(TBBT)_{12}(PDT)_3]^0$, and $[Au_{24}Pt(TBBT)_{18}]^0$ are shown for comparison. Ligand-exchange products eluted at shorter retention times than $[Au_{24}Pt(SC_6H_{13})_{18}]^0$ because TBBT is more polar than SC_6H_{13}. The fine peak progression of each major peak is caused by the existence of topological isomers.

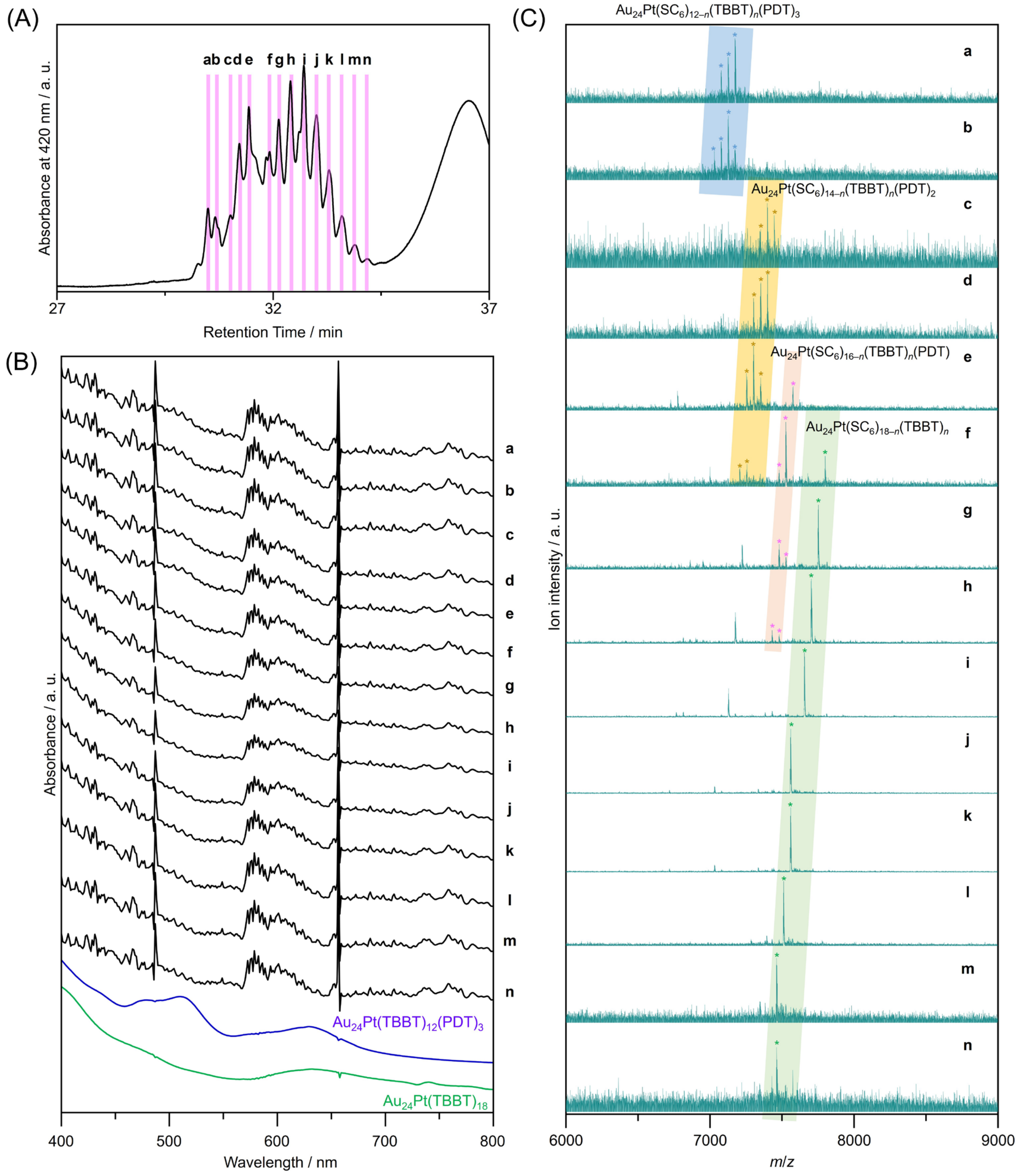

Fig. S6 (A) RP-HPLC chromatogram of products obtained following the reaction between $[Au_{24}Pt(SC_6H_{13})_{18}]^0$, $PDTH_2$, and TBBTH for 5 minutes; (B) UV-vis absorption spectra and (C) ESI-MS spectra of (a-n) in (A) obtained using the PDA detector integrated in the HPLC apparatus

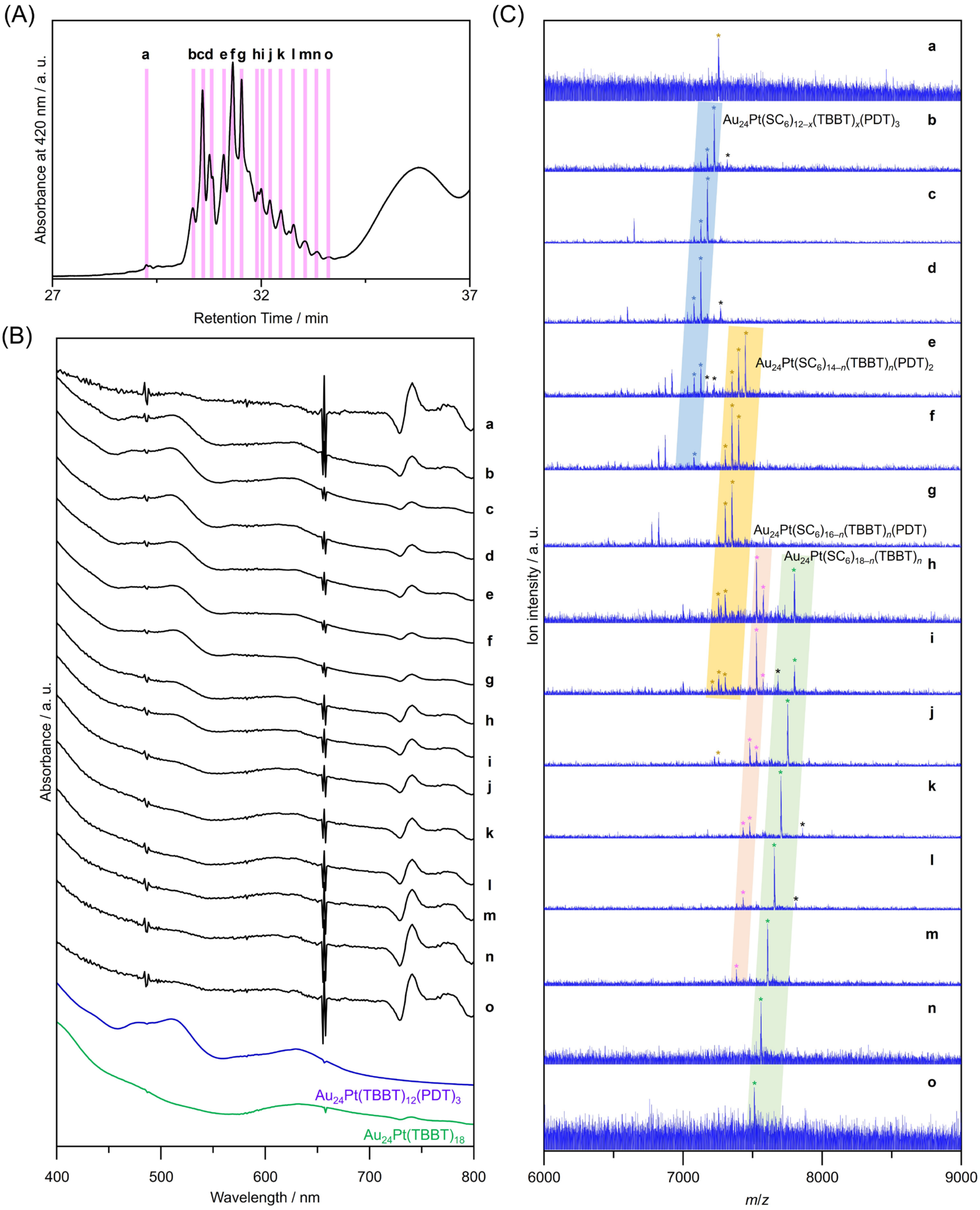

Fig. S7 (A) RP-HPLC chromatogram of products obtained following the reaction between $[Au_{24}Pt(SC_6H_{13})_{18}]^0$, $PDTH_2$, and TBBTH for 10 minutes; (B) UV-vis absorption spectra and (C) ESI-MS spectra of (a-o) in (A) obtained using the PDA detector integrated in the HPLC apparatus

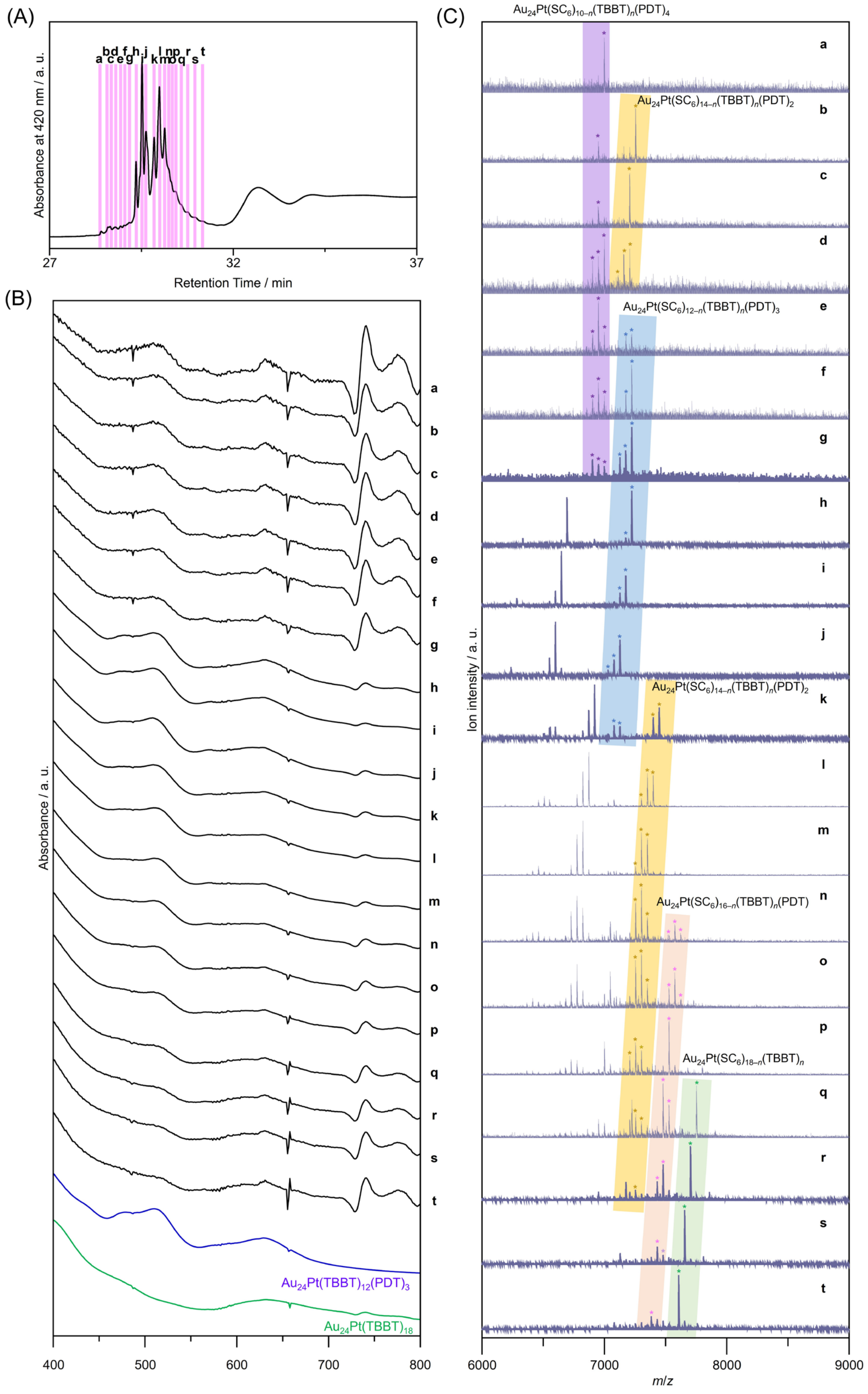

Fig. S8 (A) RP-HPLC chromatogram of products obtained following the reaction between $[Au_{24}Pt(SC_6H_{13})_{18}]^0$, $PDTH_2$, and TBBTH for 30 minutes; (B) UV-vis absorption spectra and (C) ESI-MS spectra of (a-t) in (A) obtained using the PDA detector integrated in the HPLC apparatus

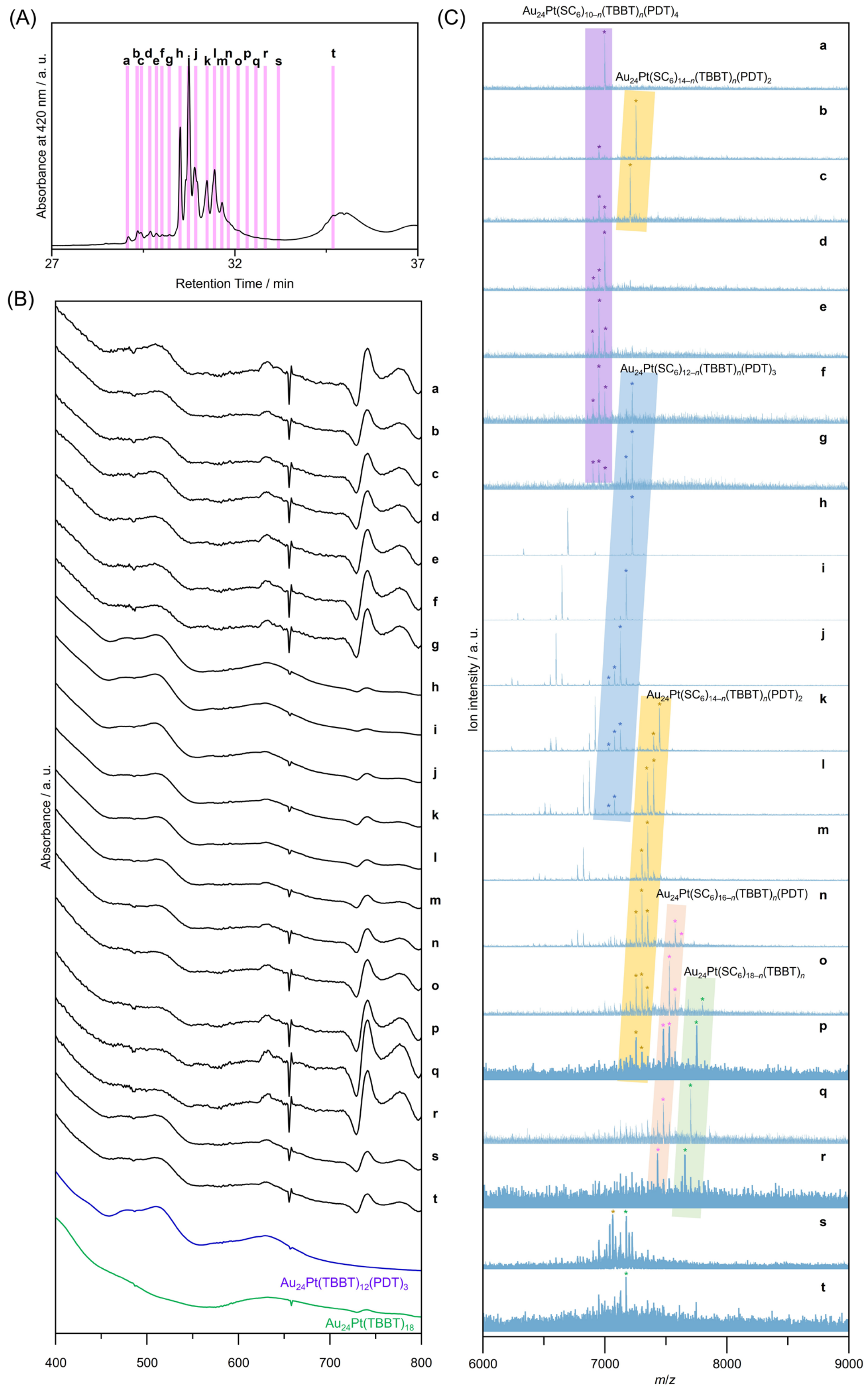

Fig. S9 (A) RP-HPLC chromatogram of products obtained following the reaction between $[Au_{24}Pt(SC_6H_{13})_{18}]^0$, $PDTH_2$, and TBBTH for 60 minutes; (B) UV-vis absorption spectra and (C) ESI-MS spectra of (a-t) in (A) obtained using the PDA detector integrated in the HPLC apparatus

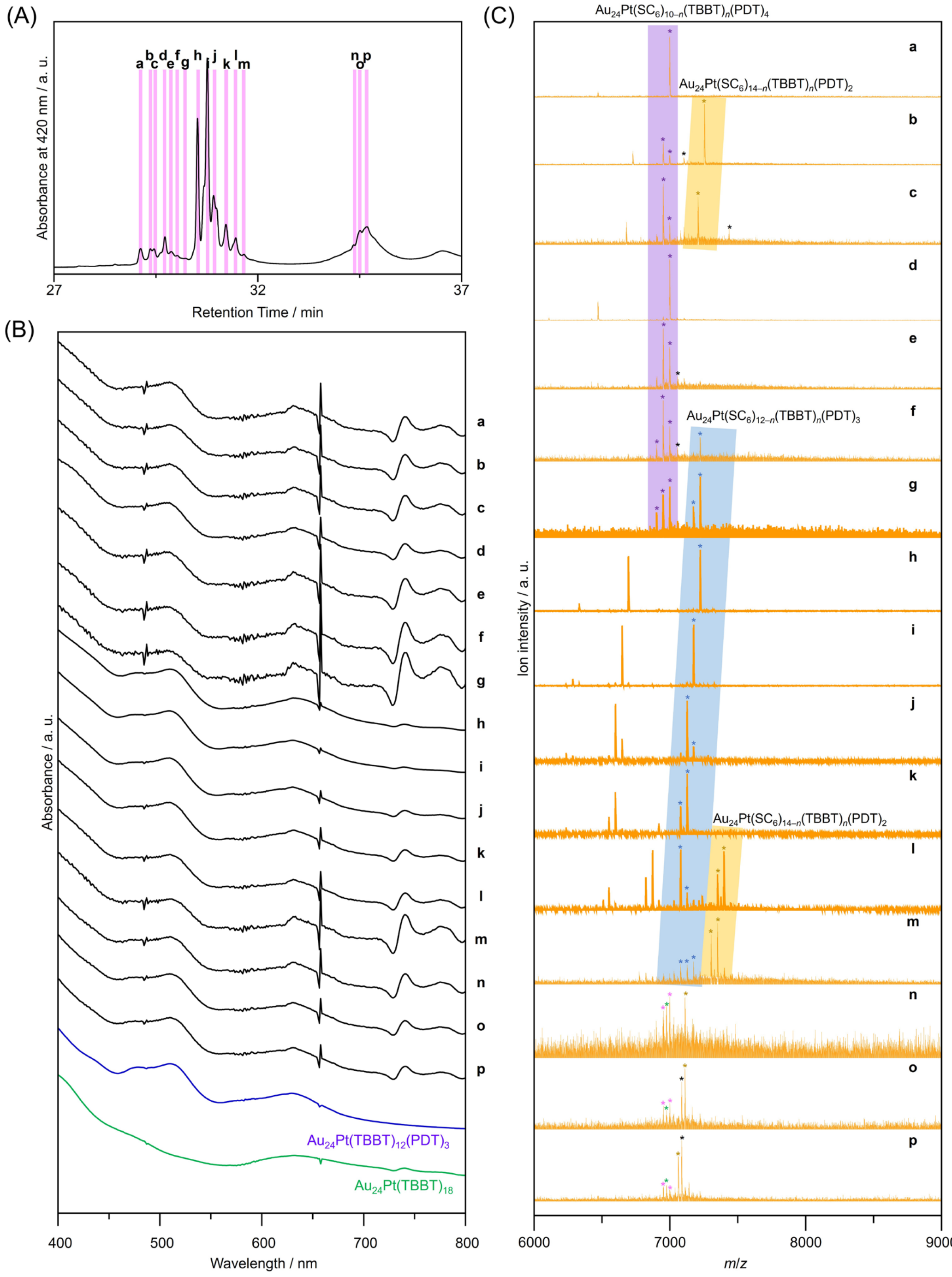

Fig. S10 (A) RP-HPLC chromatogram of products obtained following the reaction between $[Au_{24}Pt(SC_6H_{13})_{18}]^0$, $PDTH_2$, and TBBTH for 180 minutes; (B) UV-vis absorption spectra and (C) ESI-MS spectra of (a-p) in (A) obtained using the PDA detector integrated in the HPLC apparatus

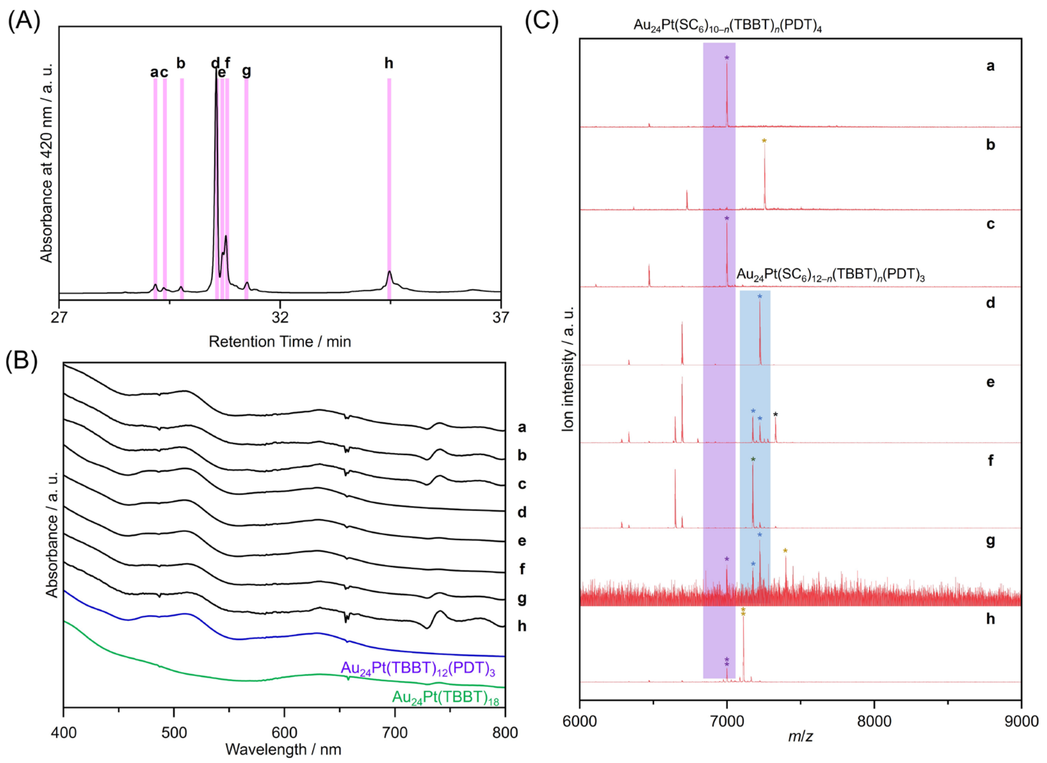

Fig. S11 (A) RP-HPLC chromatogram of products obtained following the reaction between $[Au_{24}Pt(SC_6H_{13})_{18}]^0$, $PDTH_2$, and TBBTH for 360 minutes; (B) UV-vis absorption spectra and (C) ESI-MS spectra of (a-h) in (A) obtained using the PDA detector integrated in the HPLC apparatus

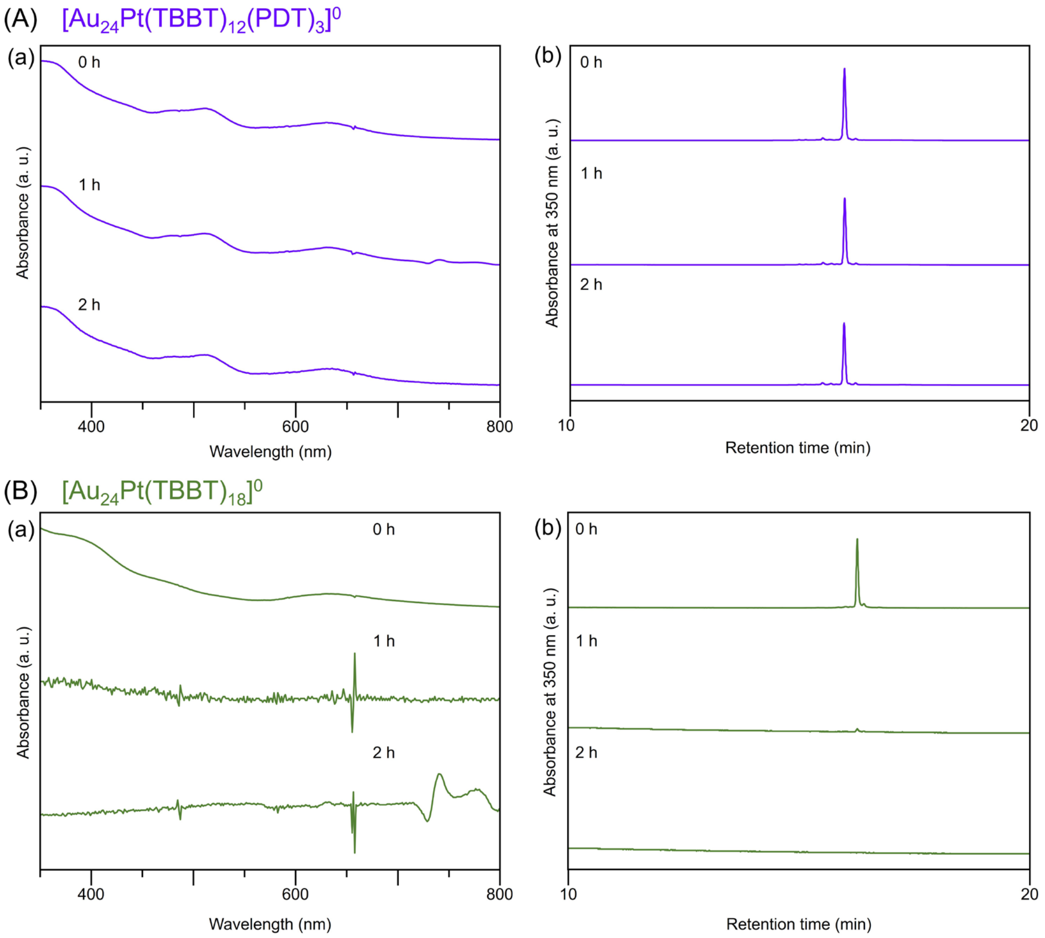

Fig. S12 Time-dependent (a) UV-vis optical absorption spectra obtained with a PDA detector attached to the HPLC apparatus and (b) RP-HPLC chromatograms for (A) $[Au_{24}Pt(TBBT)_{12}(PDT)_3]^0$ and (B) $[Au_{24}Pt(TBBT)_{18}]^0$. The NCs-dissolved solution was maintained at 80 °C in toluene, and stability was evaluated using HPLC at regular intervals. $[Au_{24}Pt(TBBT)_{12}(PDT)_3]^0$ maintained its spectrum even over extended periods at high temperatures.

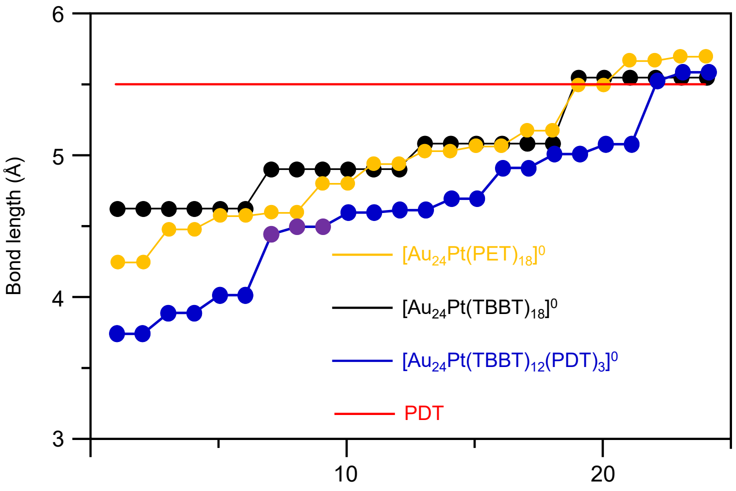

Fig. S13 S–S bond lengths in $[Au_{24}Pt(PET)_{18}]^0$, $[Au_{24}Pt(TBBT)_{18}]^0$, and $[Au_{24}Pt(TBBT)_{12}(PDT)_3]^0$; the S–S bond length in PDT is shown in red for comparison.

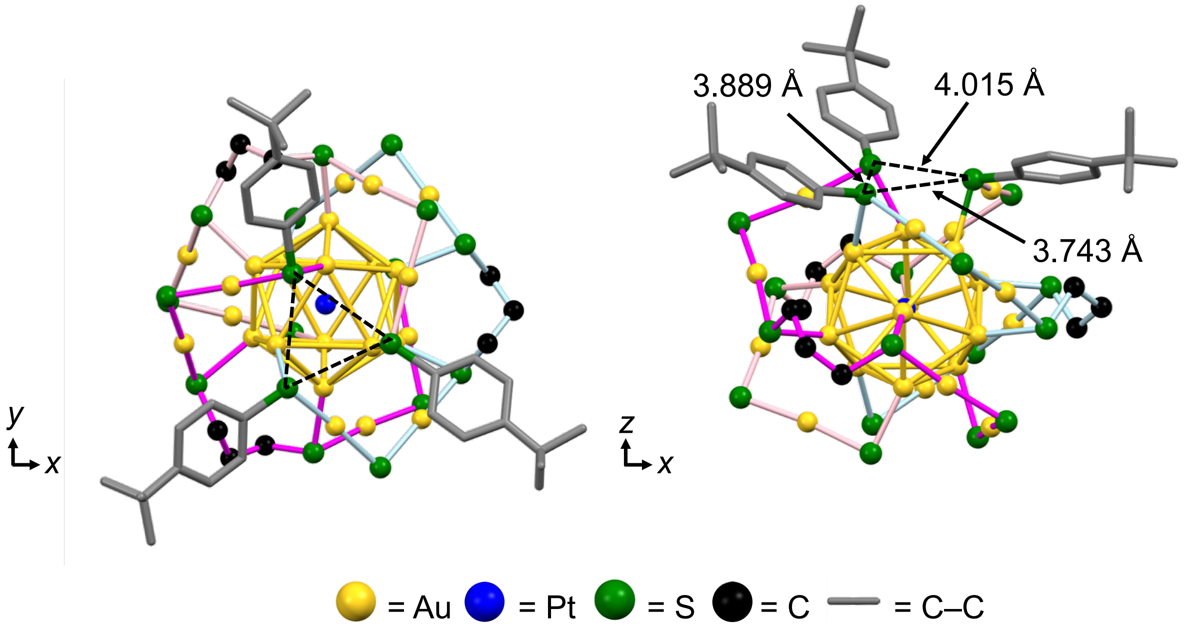

Fig. S14 Distances between inter-staple S atoms in $[Au_{24}Pt(TBBT)_{12}(PDT)_3]^0$ (dotted black line: imaginary bond). Three of six staples are omitted for better visualization/clarity. Reproduced from Ref. 1 with permission from the American Chemical Society.

S3. References

1. M. Sera, S. Hossain, S. Yoshikawa, K. Takemae, A. Ikeda, T. Tanaka, T. Kosaka, Y. Niihori, T. Kawawaki, Y. Negishi, *J. Am. Chem. Soc.* 2024, **146**, 29684-29693.
2. H. Qian, D.-e. Jiang, G. Li, C. Gayathri, A. Das, R. R. Gil, R. Jin, *J. Am. Chem. Soc.* 2012, **134**, 16159–16162
3. Y. Negishi, H. Horihata, A. Ebina, S. Miyajima, M. Nakamoto, A. Ikeda, T. Kawawaki, S. Hossain, *Chem. Sci.* 2022, **13**, 5546-5556.
4. L. M. C. Luong, X. B. Carroll, C. D. Lowe, M. M. Olmstead, A. L. Balch, *Inorg. Chim. Acta* 2022, **530**, 120682.
5. Y. Shichibu, K. Konishi, *Small* 2010, **6**, 1216-1220.
6. S. Hossain, Y. Imai, D. Suzuki, W. Choi, Z. Chen, T. Suzuki, M. Yoshioka, T. Kawawaki, D. Lee, Y. Negishi, *Nanoscale* 2019, **11**, 22089-22098.
